# Nutritional management of a kitten with thermal burns and septicaemia

**DOI:** 10.1177/2055116920930486

**Published:** 2020-06-29

**Authors:** Rachael Birkbeck, Rebekah Donaldson, Daniel L Chan

**Affiliations:** Department of Clinical Science and Services, The Royal Veterinary College, North Mymms, Hatfield, UK

**Keywords:** Hypermetabolic state, enteral nutrition, sepsis, resting energy requirement

## Abstract

**Case summary:**

A 3-month-old entire female British Shorthair cat presented for further management of thermal burns after falling into a bath of scalding water. On presentation to the primary care clinician the kitten was obtunded, markedly painful and relatively bradycardic, consistent with a state of shock. The haircoat was wet, with erythematous skin and sloughing from the digital pads and anal mucosa. The primary care clinician administered opioid analgesia, sedation, antibiotics and started intravenous (IV) fluid therapy prior to referral. On arrival to the referral hospital the kitten was obtunded with respiratory and cardiovascular stability but was overtly painful and resistant to handling. The kitten required intensive management with IV and regional analgesia, IV broad-spectrum antibiosis, IV fluid therapy, enteral nutrition and wound management, including surgical debridement and topical antibiotic therapy. Septicaemia developed during the hospitalisation. Multidrug-resistant *Escherichia coli* and *Pseudomonas aeruginosa* were cultured, and antibiosis was escalated to IV imipenem. Acute respiratory distress syndrome was suspected following the development of dyspnoea. Early enteral nutrition within 24 h of admission was initiated using an oesophageal feeding tube and a veterinary therapeutic liquid diet. Over the ensuing 72 h the kitten started voluntary intake of food alongside oesophageal tube feeds. The kitten experienced continued weight loss despite the provision of nutritional support to meet, and then later exceed, the estimated resting energy requirements. Caloric intake was gradually increased to a total of 438% of the calculated resting energy requirement using the most recent daily body weight, eventually resulting in stabilisation of weight loss and weight gain.

**Relevance and novel information:**

There is limited published information on the nutritional management of veterinary patients with thermal burn injury. Hypermetabolic states related to burn injuries are induced and maintained by complex interactions of catecholamines, stress hormones and inflammatory cytokines on proteolysis, lipolysis and glycogenolysis. Secondary infections are common following burn injury and the subsequent proinflammatory state perpetuates hypermetabolism and catabolism. These states present a challenge in both predicting and providing adequate nutrition, particularly in a paediatric septic patient. This subset of patients should be monitored closely during hospitalisation to ensure body weight and condition are maintained (while taking into consideration hydration status), and caloric intake is adjusted accordingly to meet nutritional support goals. Extensive research exists regarding the nutritional requirements and metabolic derangements of people with thermal burns. However, the importance of maintaining body weight and body condition in veterinary burn patients, and the association between nutritional support and reduced morbidity and mortality, has not been investigated and remains to be elucidated.

## Introduction

The debilitating metabolic stress response provoked by burn injury, and associated increase in energy expenditure and nitrogen loss, is unrivalled by other forms of trauma or critical illness.^1^ Management of burns is well described in human medicine, but detailed description on the subject in veterinary medicine is scarce. Beyond the challenges presented by pain, fluid resuscitation, cardiovascular instability, wound management and pulmonary disease in burn patients, a hypermetabolic state is also recognised in people, which increases the risk of patient morbidity and mortality.

Immediately after sustaining severe burn trauma, human patients have a period of decreased metabolism and reduced tissue perfusion known as the ‘ebb’ phase.^2,3^ Approximately 5 days after injury the patient enters the ‘flow’ phase, characterised by hyperdynamic circulation and a hypermetabolic phase.^3,4^ Although it will subside significantly by approximately 6 months, the hypermetabolic flow phase can persist for up to 2 years.^4,5^

The hypermetabolic state occurs as a result of the complex interaction of increased energy expenditure and inefficiency mediated by sustained catecholamine, stress hormone and inflammatory cytokine release. Increased proteolysis, lipolysis and glycogenolysis occur, and metabolic alterations predominantly affect the liver, adipose tissue and skeletal muscle.^3^

Failure to recognise and treat burn trauma hypermetabolism results in accelerated substrate turnover and muscle wasting, which may contribute to increased morbidity and mortality.^4,6,7^ To our knowledge, management of a hypermetabolic state has not been described in a veterinary burn patient.

## Case description

A 3-month-old entire female British Shorthair cat was referred to the Queen Mother Hospital for Animals for further management of thermal burns. The kitten had fallen into a bath of scalding water for approximately 30 s before she was removed, and first aid was administered by placing her in cold water for approximately 10 mins. In the UK, hot water is stored at 60^°^C to kill *Legionella* species. Although the temperature of the scalding bath water is not known, given that the bath was being filled exclusively with hot water the temperature was likely to have been between 45ºC and 55ºC.

The kitten presented to the primary care practice obtunded, vocalising and shivering. The haircoat was wet, with erythematous skin and sloughing from the digital pads and anal mucosa. The respiratory rate was 20 breaths per min, heart rate was 170 beats per min and mucous membranes were pink and moist, with a capillary refill time <2 s. Thoracic auscultation was unremarkable. The kitten was assessed as euhydrated. The rectal temperature was 37.9ºC. Severe pain limited further examination. Methadone was administered intramuscularly (IM) at 0.3 mg/kg (Synthadon; Animalcare).

The kitten was sedated with medetomidine at 25 µg/kg IM (Domitor; Vetoquinol) to facilitate intravenous (IV) catheter placement. The level of sedation was insufficient after 15 mins and a second dose of medetomidine at 20 µg/kg IM was administered, which provided suitable sedation for IV catheter placement. Following IV placement, cefuroxime 15 mg/kg IV (Zinacef; GlaxoSmithKline) was administered. IV isotonic crystalloid therapy was initiated at 6 ml/kg/h (compound sodium lactate; Aquapharm) for approximately 2 h prior to referral.

The kitten presented to the referral hospital approximately 6 h after sustaining the burn trauma. On arrival, the kitten was obtunded, overtly painful, vocalising and resistant to handling. An IV fentanyl bolus (Fentadon; Dechra) of 2 µg/kg was administered and continued as a constant rate infusion (CRI) at 4 µg/kg/h. Limited physical examination confirmed respiratory and cardiovascular stability. Measurement of blood pressure and rectal temperature were not tolerated owing to pain and distress. The kitten was euhydrated and weighed 1.53 kg with a body condition score (BCS) of 4/9 and muscle condition score of 2/3.

Initial point-of-care venous blood gas and metabolite analysis identified mild hyperglycaemia attributed to stress (8.5 mmol/l; reference interval [RI] 4.7–7.3 mmol/l [156.6 mg/dl; RI 84.6–131.4 mg/dl]); all other results were within the RIs (Table 1). IV isotonic crystalloid therapy (compound sodium lactate) was restarted at 6 ml/kg/h. Throughout hospitalisation, IV potassium chloride (Hameln) was supplemented as required by adding it into the crystalloid fluid bag.^8^

**Table 1:** Summary of serial haematological, biochemical and venous blood gas analyses during the hospitalisation period

Hospitalisation day	1	4	6	18	Range
Haematology					
WBC (×10^9^/l)	3.21	5.78	6.81	39.31	5.5–19.5
Neutrophils (×10^9^/l)	1.41	3.87	3.00	25.16	2.5–12.5
Haematocrit (%)	20.4	18.3	13.4	15.3	24–45
Reticulocytes (%)	–	0.2	1.6	2.0	–
Platelets (×10^9^/l)	>200	>240	>340	60	200–800
Biochemistry					
Albumin (g/l)	17.3	17.3	–	–	25–45
Cholesterol (mmol/l)	1.68	2.60	–	–	2.2–4
Alanine aminotransferase (U/l)	231.7	171.6	–	–	5–60
Creatine kinase (U/l)	1340	1830	–	–	57–574
Creatinine (µmol/l)	23	17	29	–	20–177
Total bilirubin (µmol/l)	2.4	2.2	0	0	0.1–5.1
Magnesium (mmol/l)	0.62	–	–	–	0.8–1.2
Lipaemia index	None	None	None	None	
Electrolyte and blood gas analysis					
pH	7.272	7.411	7.443	7.358	7.350–7.470
pvCO_2_ (mmHg)	37.5	28.8	29.0	46.5	37.0–47.0
Base excess (mmol/l)	–8.8	–5.8	–3.9	0.6	–
Glucose (mmol/l)	8.5	11.5	9.7	8.1	4.7–7.3
Lactate (mmol/l)	1.5	1.2	–	0.6	0.6–2.5
Sodium (mmol/l)	143	143	146	156	140–153
Potassium (mmol/l)	3.5	4.2	4.2	3.6	3.6–4.6
Chloride (mmol/l)	121	111	114	119	106–120

Venous blood gas analyses performed on subsequent days are not shown as no extraneous values were documented

WBC = white blood cell count

A modified Colorado State University Medical Center Feline Acute Pain Scale score of 9/12 prompted escalation of analgesia. IV ketamine (Anaestamine; Animalcare) 0.5 mg/kg followed by 0.2 mg/kg/h CRI and IV medetomidine (Medetor; Virbac) 0.1 µg/kg followed by 0.5–1 µg/kg/h CRI were introduced sequentially to achieve a pain score of 2–4/12. This multimodal analgesia protocol was continued for 3 days and then gradually weaned as guided by serial pain scoring.

**Figure 1:**
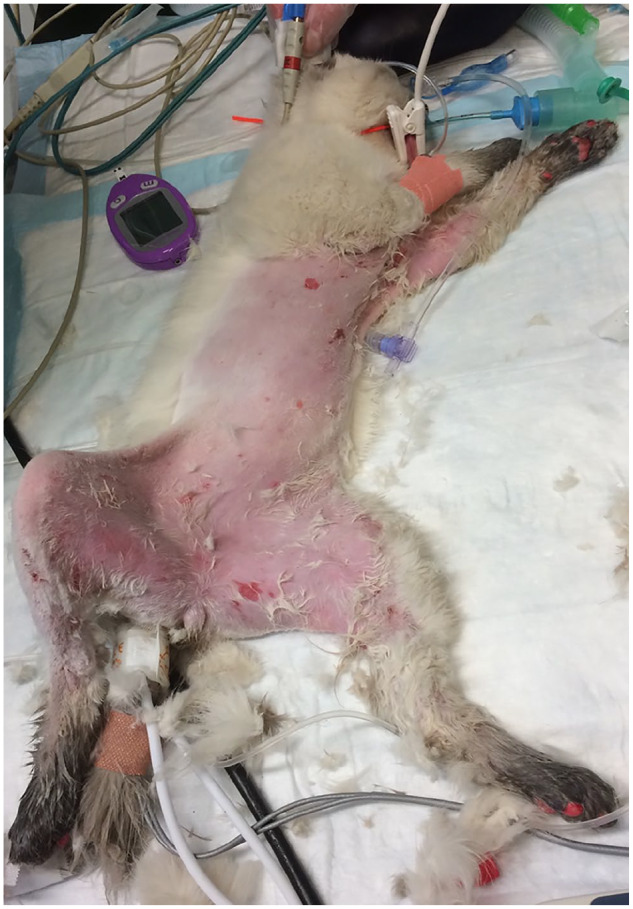
Clipping of the haircoat following admission revealing scalding and erythema of the ventral thorax, abdomen, thoracic/pelvic limbs and perineum with areas of ulceration

General anaesthesia was performed following initial stabilisation within 2 h of admission. The kitten was premedicated with midazolam 0.2 mg/kg IV (Hyponovel; Roche) and anaesthesia was induced with propofol (PropoFlo; Abbot) administered incrementally to effect (total dose 2.5 mg/kg). Anaesthesia was maintained with isoflurane delivered in 100% oxygen. The haircoat was clipped, revealing scalding and erythema of the ventral thorax, abdomen, thoracic and pelvic limbs, and perineum, with areas of ulceration (Figure 1). The head, neck, oral cavity and corneas were unaffected.

The extent of thermal injury was approximated to be 80% of the total body surface area (TBSA) based on the ‘rule of nines’.^9,10^ The burn injury was classified as deep partial thickness (second degree).^11^ Ulcerated areas were cleaned with dilute iodine; antimicrobial cream was not applied as the lesions were small and superficial. The patient was instrumented with a central venous catheter, urinary catheter and oesophagostomy feeding tube (O-tube); correct placement was confirmed with thoracic radiography. The O-tube was placed as described by Mazzaferro,^12^ and the insertion stoma was covered with a sterile dressing and checked twice daily for signs of infection. Pyrexia of 39.7ºC was documented after the kitten had recovered from general anaesthesia.

**Figure 2:**
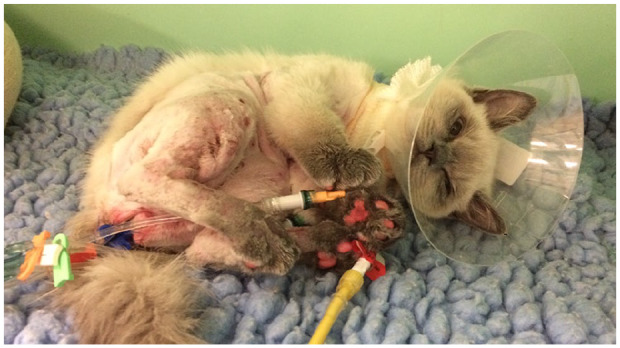
Enteral nutrition was administered by constant rate infusion via the oesophageal feeding tube

Prior to sustaining the thermal burn injury, the kitten was being fed a mixture of complete and balanced wet/dry kitten foods appropriate for growth. Daily resting energy requirement (RER) was calculated to be 96 kcal/day using the formula RER = 70 × body weight (kg)^0.75^. The most recent daily weight was used to calculate the daily RER during hospitalisation. A veterinary liquid diet (Gastrointestinal High Energy; Royal Canin) was fed as a CRI within 12 h of admission at 50% of the kitten’s calculated RER (48 kcal). The kitten initially showed no interest in voluntary intake of food and provision of enteral nutrition via the O-tube was incrementally increased to 100% (96 kcal/day) within the first 48 h (Figure 2).

The therapeutic liquid diet (Gastrointestinal High Energy; Royal Canin) was chosen for ease of administration and high calorie content. It was used for all O-tube feeds and administered as a CRI. Although marketed for dogs, this liquid diet met the minimum nutritional content recommended for growing kittens for the majority of nutrients (Table 2). The protein, calcium and phosphorus levels of this diet were slightly below the minimum content recommended for growing kittens. In order to mitigate this deficiency, the kitten was also offered a canned wet food diet specifically formulated to support growth in cats (Hill’s Science Plan Kitten) at six intervals throughout the day.

**Table 2:** Comparison of minimum recommended dietary nutrient requirement for feline growth stage and diets fed during hospitalisation

Nutrient content	Minimum recommended for feline growth stage*	O-tube (Gastrointestinal High Energy; Royal Canin)	Voluntary intake (Hill’s Science Plan Kitten)
Macronutrient			
Protein (g/100 kcal)	7	6.11	11.4
Fat (g/100 kcal)	2.25	4.75	5.5
Carbohydrate (g/100 kcal)	No recommendation	7.94	3.8
Fatty acids			
EPA + DHA (g/100 kcal)	<0.01	0.09	0.06
Amino acids			
Arginine (g/100 kcal)	0.27	0.68	0.53
Taurine (g/100 kcal)	0.06	0.14	0.09
Minerals			
Calcium (g/100 kcal)	0.20	0.19	0.30
Phosphorus (g/100 kcal)	0.21	0.18	0.26
Magnesium (g/100 kcal)	0.01	0.01	0.03
Sodium (g/100 kcal)	0.04	0.13	0.07
Potassium (g/100 kcal)	0.15	0.20	0.21
Iron (mg/100 kcal)	2	2.71	NA
Copper (mg/100 kcal)	0.25	0.28	NA
Zinc (mg/100 kcal)	1.88	4.07	NA
Selenium (mg/100 kcal)	7.5	20.35	NA
Vitamins			
Vitamin A (IU/100 kcal)	225	339.21	2366
Vitamin D3 (IU/100 kcal)	7	32.56	19
Vitamin E (mg/100 kcal)	0.95	16.96	14
Vitamin C (mg/100 kcal)	No recommendation	13.57	1.6
Vitamin B1 (mg/100 kcal)	No recommendation	0.13	NA
Vitamin B2 (mg/100 kcal)	No recommendation	0.31	NA
Vitamin B6 (mg/100 kcal)	0.06	0.08	NA
Niacin (mg/100 kcal)	0.8	1.36	NA
Pantothenic acid (mg/100 kcal)	0.14	0.68	NA
Vitamin B12 (µg/100 kcal)	0.45	1.76	NA
Folic acid (mg/100 kcal)	0.02	0.08	NA
Biotin (µg/100 kcal)	<0.01	5.43	NA
Choline (mg/100 kcal)	80	67.84	NA
Energy	Not applicable	1.5 kcal/ml	1.20 kcal/g

* The European Pet Food Industry (www.fediaf.org)

EPA = eicosapentaenoic acid; DHA = docosahexaenoic acid; NA = not available – manufacturer declined to provide information

Prior to planned anaesthesia and surgical procedures, the kitten received a final feed the night before at 2 am, after which food was withheld and the O-tube CRI stopped. Feeding was restarted once the kitten had recovered from anaesthesia and the frequency of feeds and CRI volume adjusted to ensure the daily calorie target was met. The kitten remained euhydrated based on clinical examination throughout hospitalisation; therefore, fluctuations in body weight were not attributed to changes in fluid balance.

Haematology and biochemistry panels sampled on admission revealed a non-regenerative mild anaemia, neutropenia, hypoalbuminaemia, hypocholesterolaemia and increased alanine aminotransferase (ALT) activity (Table 1). On day 2, the kitten remained pyrexic at 39.9ºC. The urinary catheter was removed and the central venous catheter was replaced as it had been removed by the patient. Pyrexia persisted despite new vascular access. No erythema or discharge of the O-tube site was detected on daily assessment. Urinalysis indicated no evidence of urinary tract infection and urine culture demonstrated no microbial growth. IV potentiated amoxicillin 20 mg/kg q8h (Augmentin; GlaxoSmithKline) was initiated on day 3.

**Figure 3:**
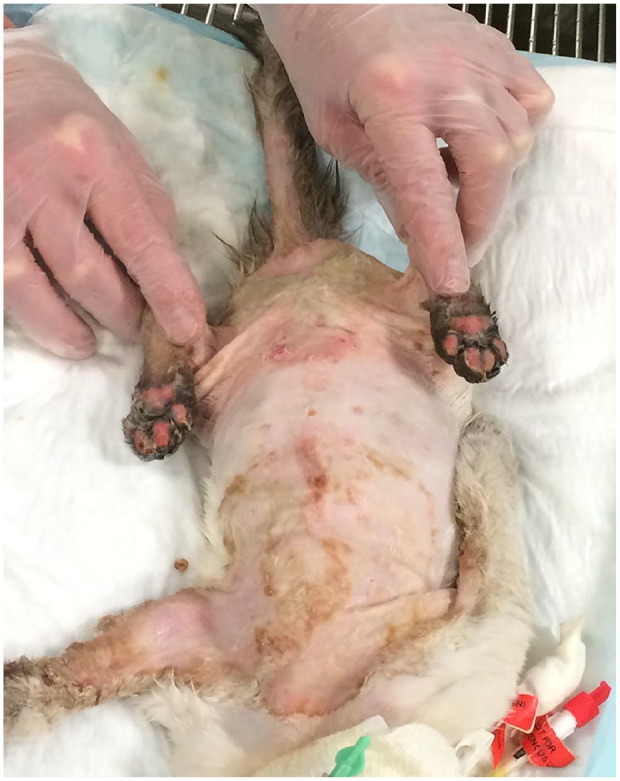
Progression of the deep partial thickness (second degree) burns on day 4 of hospitalisation

On day 3, the kitten started to eat small quantities of kitten food (Hill’s Science Plan Kitten) when offered by hand. The calories ingested voluntarily were minimal and O-tube feeding continued at 100% of the calculated RER. Neutropenia resolved on day 4 (Table 1); however, the pyrexia persisted, with the rectal temperature fluctuating between 39.3ºC and 40.5 °C. In the absence of documented infection, the pyrexia was attributed to severe systemic inflammation. Antibiotics were continued due to the severity of the skin lesions (Figure 3). Biochemistry revealed static hypoproteinaemia, hypomagnesaemia, improved ALT and increased creatine kinase activities (Table 1). Magnesium was not supplemented or reassessed in the absence of clinical signs or associated electrolyte derangements consistent with hypomagnesaemia. Blood glucose remained mildly increased throughout hospitalisation (Table 1).

**Figure 4:**
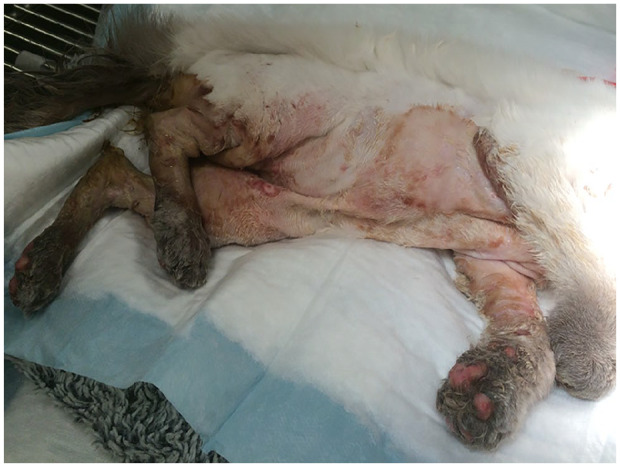
Necrosis of skin lesions prior to debridement on day 8 of hospitalisation

Between admission and day 4, the kitten lost 80 g (5.2% body weight loss), decreasing to 1.45 kg. On day 4, the kitten received a total of 120 kcal (130% of its calculated RER for the most recent daily body weight), of which 36 kcal (30%) was voluntary intake of kitten food. By day 6, the kitten’s calorie intake increased to 240 kcal/day (260% of the calculated RER for the most recent daily body weight), 120 kcal (50%) of which was achieved via voluntary intake. The kitten’s appetite continued to improve and by day 7 it was eating 96 kcal/day (104% of its calculated RER), and also tolerating 144 kcal/day (157% of calculated RER) via the O-tube. Between days 4 and 8 of hospitalisation, the kitten’s body weight decreased further to 1.40 kg (3.5% body weight loss). On day 8 of hospitalisation, general anaesthesia was planned to facilitate wound debridement (Figure 4); thereafter, silver sulfadiazine cream (Flamazine; Smith&Nephew) was applied daily. On day 9, voluntary food intake had increased to 120 kcal/day (133% of calculated RER using the most recent daily body weight), which was matched by O-tube feeding to bring the total daily calorie intake to 240 kcal/day (266% of the calculated RER).

On day 11, general anaesthesia was performed to facilitate extensive debridement of the more severely affected left inguinal area and hindlimb, and to perform an epidural consisting of 0.1 mg/kg morphine (Morphine Sulfate; Macarthys Laboratories) and 1.5 mg/kg ropivacaine 0.25% (Naropin; AstraZeneca) to provide regional anaesthesia postoperatively. This enabled de-escalation of systemic analgesia from fentanyl 1–5 µg/kg/h CRI to methadone 0.1–0.2 mg/kg IV q4h (Comfortan; Dechra). On day 13, buprenorphine (Buprecare; Animalcare) 0.01–0.02 mg/kg IV q6h, gabapentin (Gabapentin; Milpharm) 8 mg/kg PO q12h and meloxicam (Metacam; Boehringer Ingelheim) 0.05 mg/kg IV q24h were introduced.

Throughout days 9–12 of hospitalisation, the kitten’s total calorie intake was gradually increased. However, voluntary intake did not exceed 120 kcal/day (133% of the calculated RER), despite the kitten being offered in excess of this amount. As such, in order to increase voluntary intake further, 4 g of veterinary therapeutic instant diet powder (Convalescence Support; Royal Canin) was added to all meals offered. This increased voluntary calorie intake to approximately 145 kcal/day (161% of the calculated RER), with a minimal increase in the volume of food ingested. In addition, 225 kcal/day (250% of the calculated RER) was provided via the O-tube CRI. This resulted in the kitten receiving a total of 315 kcal/day (350% RER) by day 12. Despite increased nutritional provision, between days 8 and 12 of hospitalisation the kitten lost a further 50 g of body weight (3.6% body weight), decreasing to 1.35 kg.

On day 14 of hospitalisation, a new heart murmur was documented. Echocardiography did not reveal underlying structural disease or changes consistent with endocarditis. The murmur was deemed to be haemic secondary to anaemia (Table 1). Cardiac troponin I was mildly increased (0.4 ng/ml; RI <0.04). The mild increase in cardiac troponin I concentration was attributed to myocardial injury related to systemic inflammation.

While hospitalised, the kitten received IV fluid therapy (IVFT) at 5–6 ml/kg/h based on regular assessment of hydration status, fluid intake and losses. On day 13, IVFT was decreased from 5 ml/kg/h to 3 ml/kg/h. The following day, IVFT was reduced further to 1 ml/kg/h before being discontinued on day 15. Fluid therapy was discontinued as clinical assessment indicated that enteral fluid intake was sufficient to meet the kitten’s needs and significant losses were not occurring.

Dyspnoea developed on day 15 of hospitalisation and the kitten was placed in an oxygen kennel set at 60% fraction of inspired O_2_. Thoracic radiographs identified cardiomegaly without infiltrative pulmonary disease. Point-of-care ultrasound identified diffuse pulmonary B lines on day 16 of hospitalisation. Pulmonary B lines are associated with peripheral interstitial–alveolar disease of varying aetiology.^13^ Acute respiratory distress syndrome was suspected, although thromboembolism or aspiration pneumonia could not be excluded. The respiratory signs resolved within 7 days of occurring and oxygen therapy was de-escalated.

The kitten remained persistently pyrexic since admission. On day 16, septicaemia was diagnosed when repeat haematology documented phagocytosis of bacterial cocci. Blood cultures were obtained prior to escalation of antibiosis. The kitten had been receiving potentiated amoxicillin (Augmentin; GlaxoSmithKline) 20 mg/kg q8h since day 3 of hospitalisation. IV imipenem (Primaxin; Merck Sharp & Dohme) 10 mg/kg q8h was introduced concurrently pending blood culture results. Growth of multidrug-resistant *Escherichia coli* and *Pseudomonas aeruginosa* (resistant to potentiated amoxicillin) was reported after 48 h incubation from enrichment culture. Both isolates were susceptible to imipenem, which was continued for a total of 5 days. Potentiated amoxicillin was discontinued once the culture results had been received. The kitten’s pyrexia resolved within 24 h of initiating imipenem (Primaxin; Merck Sharp & Dohme).

Throughout days 12–17 of hospitalisation, the kitten’s total calorie intake was increased to 385 kcal/day (438% of the calculated RER) by increasing the calories fed via the O-tube. Calorie consumption is reported in relation to the kitten’s RER for ease of comparisons throughout this paper as the RER calculation is only dependent on body weight. On day 17, the O-tube was prematurely removed by the patient. The kitten was able to maintain a voluntary calorie intake of 289 kcal/day (328% of the calculated RER) and its weight remained static. Between days 12 and 24, the kitten’s body weight gradually increased by 50 g to 1.4 kg (3.6% of its current body weight). Cessation of weight loss indicated that its apparent energy requirements were being met.

The kitten was discharged from the hospital on day 24. Dietary recommendations at discharge were to feed a minimum of 289 kcal/day (328% of the calculated RER) of a complete and balanced growth diet (Hill’s Science Plan Kitten) and increase the amount of food offered if there was a decrease in body weight. Body weight was 1.4 kg vs 1.53 kg on admission. BCS was 3/9 vs 4/9 on admission and the muscle condition score had decreased from 2/3 to 1/3.

**Figure 5:**
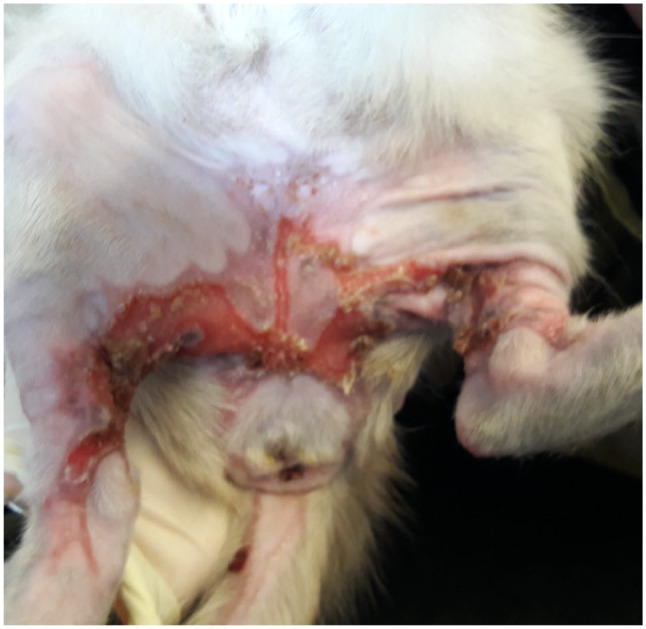
Incomplete healing of skin lesions 5 days after discharge from the hospital

There was incomplete healing of the thermal burns at discharge. Medication dispensed included sublingual buprenorphine 0.02 mg/kg q8h (Buprecare; Animalcare), oral meloxicam (Metacam; Boehringer Ingelheim) 0.05 mg/kg q24h and gabapentin (Gabapentin; Milpharm) 8 mg/kg q12h, to be discontinued at the discretion of the primary care veterinary surgeon. Clinical reassessment was performed at the primary care practice 5 days after discharge (Figure 5).

At follow-up 18 months post-injury the kitten weighed 3.97 kg with a BCS of 4/9 and muscle condition score of 2/3. In addition, there had been complete healing of the deep partial-thickness burns and full regrowth of hair.

## Discussion

This case report is the first to detail nutritional management of a paediatric feline patient with extensive deep partial-thickness (second-degree) thermal burns, with caloric intake provided far in excess of calculated RER to maintain body weight. Precise measurement of a patient’s energy requirement using indirect calorimetry is only available in a select number of veterinary hospitals. In clinical practice the allometric formula RER (kcals/day) = 70 × (current body weight in kg)^0.75^ is widely used to estimate RER.^14^ Paediatric patients have increased energy requirements owing to growth, and they are therefore more likely to be affected by hypermetabolic states and are at a higher risk of malnutrition.^15^

Because ongoing loss of weight and body condition occurred initially despite increased caloric provision, a hypermetabolic state was assumed. In people, a hypermetabolic state is diagnosed when the resting energy expenditure is increased by more than 10%.^16^ In the acute post-burn injury phase, energy expenditure increases proportionally with burn size, and patients with >40% of TBSA burns can have a resting energy expenditure between 40% and 80% above normal.^4,17,18^

Catecholamine release mediates hypermetabolism by inducing a significant and persistent stress response in burn trauma patients.^19–22^ In people, up to approximately 50% of the hypermetabolic response to burn injury is attributed to increased resting energy expenditure due to catecholamine-induced adenosine triphosphate consumption for gluconeogenesis, protein synthesis and urea production.^23^ Persistent hyperglycaemia owing to insulin resistance is induced by catecholamines, hypercortisolaemia, cytokines and downregulation of glucose transporter type 4 (GLUT4) in skeletal muscle.^24^ Altered hepatic metabolism is maintained by increased circulating interleukin (IL)-6, inducing an acute-phase response in the liver.^25^ Lipolysis occurs, but suppression of beta oxidation prevents lipid utility for energy and results in hepatic lipidosis.^26^ The following burn trauma proteolysis exceeds protein synthesis and leads to skeletal muscle wasting.^6,27^

**Figure 6:**
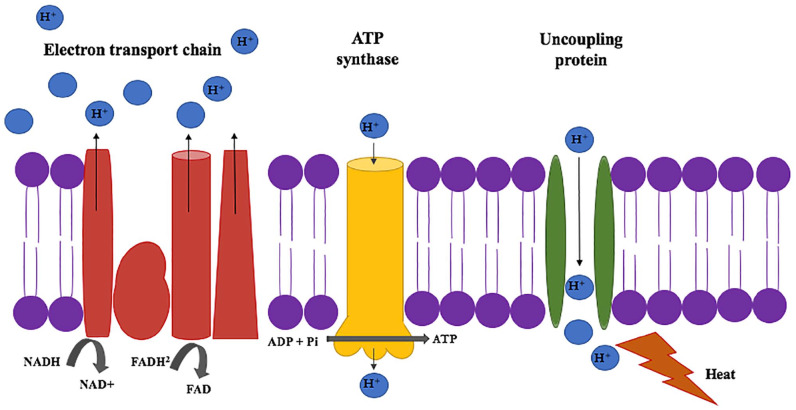
Electron transport chain. Protons are pumped from the inner mitochondrial membrane into the intermitochondrial space to create a concentration gradient. Adenosine triphosphate (ATP) synthase: the flow of hydrogen ions down the concentration gradient through ATP synthase activates catalytic sites and joins inorganic phosphate to adenosine diphosphate (ADP), resulting in the production of ATP. Uncoupling protein: permits passive movement of hydrogen ions across the mitochondrial membrane down the transmembrane proton gradient. The energy potential of the proton gradient is not used to produce ATP and is instead dissipated as heat. NADH = nicotinamide adenine dinucleotide; FADH = flavin adenine dinucleotide

Further increases in energy expenditure in burn-associated hypermetabolism are thought to be mediated by uncoupling protein 1 (UCP1) (Figure 6).^16,23,28–31^ UCP1 is a mitochondrial membrane protein usually found in mammalian brown adipose tissue that facilitates non-shivering thermogenesis.^32,33^ Experimental work in murine models of severe burn trauma have demonstrated that UCP1 mRNA is upregulated in white adipose tissue post-injury.^29,34^ Evidence suggests that conditions associated with adrenergic stress, such as severe thermal injury, induce UPC1-mediated thermogenesis in white adipose tissue and increase thermogenesis in functional brown adipose tissue.^16–18^ In patients with burn trauma the proportion of energy used for ATP production is decreased, and the proportion of energy used for thermogenesis is increased, creating an energy deficit.^16^

Sepsis further perpetuates the hypermetabolic state by driving catecholamine, stress hormone and inflammatory cytokine release.^35^ Burn patients are more susceptible to sepsis owing to immune dysfunction and disruption of the natural protection provided by the epithelium.^36–40^ Burn patients often require instrumentation with IV catheters and feeding tubes, which increase infection risk. Wound healing is also impaired as overproduction of IL-1, IL-2, IL-3 and tumour necrosis factor alpha leads to defective macrophage activity, impaired leukocyte chemotaxis, and fibroblast and epidermal cell dysfunction.^36–38,40^

Early nutritional support should be initiated once the patient is cardiovascularly stable, euhydrated and significant acid–base and electrolyte abnormalities have been corrected. It is important when introducing nutritional support that the calorie intake is incrementally increased over 24–48 h to ensure feeding is tolerated by the patient. The application of ‘illness factors’ to standardise increased nutritional demand of patients with severe illness or injury is no longer favoured as overnutrition is associated with metabolic and gastrointestinal complications, hepatic dysfunction and increased CO_2_ production.^41,42^ It is recommended that the nutritional plan is tailored to the individual patient and the energy intake increased by an additional 25–50% of the calculated RER if there is a decline in body weight or BCS.^41–43^ As a reference point, the maintenance energy requirement (MER) of healthy animals is the RER × activity factor (encompassing thermogenesis, spontaneous activity and exercise), which typically ranges between 1.1 and 2.0.^42^ Studies evaluating energy expenditure in hospitalised dogs have demonstrated this to be less than the MER.^44,45^ To our knowledge, MER has not been determined in hospitalised cats or kittens. In the case described, the activity levels of the kitten remained low throughout hospitalisation. The kitten’s weight did not stabilise until later in the course of hospitalisation, when caloric provision was >328% the RER and, on occasion, even reached 438% RER. Frequent (ie, daily) body weight assessment may help with better nutritional planning of energy requirements in hospitalised animals.

Enteral nutrition is preferred over parenteral nutrition in burn patients and feeding tube placement may be required in anorexic patients or to supplement voluntary intake. In people, enteral nutrition is associated with significantly fewer septic and metabolic complications, and significantly lower mortality rates.^46,47^ Concurrent administration of parenteral nutrition to meet estimated energy needs can be considered if patients are unable to tolerate the volume of enteral nutrition required to meet their calculated RER, or have maldigestion or malassimilation syndromes.

No standardised nutritional guidelines outlining dietary composition for veterinary burn patients exist. The European Society for Clinical Nutrition and Metabolism recommends that 50–60% of the energy requirement of an adult human burn patient is met by the provision of carbohydrates, which is not to exceed 7 g/kg/day.^48^ Judicious carbohydrate provision is required as excessive carbohydrates cause hyperglycaemia, while inadequate carbohydrate provision drives uncontrolled protein catabolism. Provision of protein comprising 20–25% of energy requirements has been recommended in adults; however, for paediatric burn patients the provision of higher protein at 1.5–3.0 g/kg/day is advised.^48^ There are no specific recommendations for dietary lipid; however, it is suggested that energy from fat should be <35% of the total energy intake.^48^ Increased provision of vitamin C, vitamin D_3_, zinc, copper and selenium, which are immune-modulating nutrients, is recommended in human burn patients.^49^

The combination of diets fed ensured that the kitten received adequate protein and significantly increased vitamin C, D_3_ and selenium compared with the minimum recommended amounts for the feline growth stage (Table 2). However, carbohydrate provision exceeded recommendations in human patients and may have contributed to the observed hyperglycaemia (Table 1). In people, the use of anabolic agents such as insulin and beta-adrenergic blockers to minimise the proinflammatory response and mitigate hyperglycaemia is reported.^15,20,48,50^ Veterinary literature regarding the use of these agents in burn injury is lacking. In people with thermal burns, glycaemic control is advised when blood glucose exceeds 8 mmol/l.^48^ Although persistent mild hyperglycaemia was documented, the kitten’s size limited serial blood sampling for glucose monitoring. Attachment of a continuous interstitial glucose monitoring device was not possible owing to cutaneous injury. As such, strict glycaemic control was not targeted owing to the risk of hypoglycaemia.

## Conclusions

Hypermetabolism following burn injury presents a challenge in both predicting and providing adequate nutrition, particularly in a septic paediatric patient. This subset of patients require close monitoring during hospitalisation to ensure body weight is maintained, with consideration given to concurrent hydration status. Caloric intake should be adjusted to meet nutritional support targets.
